# Reproductive Morphometric Plasticity in *Gregarina polymorpha* Infecting *Tenebrio molitor* Larvae

**DOI:** 10.1111/jeu.70110

**Published:** 2026-07-31

**Authors:** Alejandro Javier Correa‐Espinosa, Gregorio Pérez‐Cordón

**Affiliations:** ^1^ Department of Parasitology, Faculty of Pharmacy University of Granada Granada Spain

**Keywords:** Apicomplexa, cryptic diversity, developmental variability, phenotypic plasticity, reproductive morphology, SSU rRNA

## Abstract

Morphological variability remains one of the principal challenges in the taxonomy and species delimitation of gregarines (Apicomplexa: Gregarinomorphea). In the present study, we investigated morphometric variability in gametocysts and syzygies of *Gregarina polymorpha* infecting *Tenebrio molitor* larvae through quantitative morphometric and molecular analyses. A total of 100 gametocysts and 100 independent syzygies were examined using light microscopy and digital morphometry. Gametocyst size exhibited extensive variability, with estimated areas ranging from 4850 to 30,844 μm^2^ and coefficients of variation exceeding 30%. Size distributions significantly departed from normality and displayed marked heterogeneity associated with the presence of unusually small gametocysts. However, Hartigan's dip test did not detect statistically significant deviation from unimodality. Within syzygies, primite and satellite areas were strongly positively correlated (Pearson's *r* = 0.726, *p* < 0.001), and standard major axis regression indicated growth patterns consistent with approximate isometry despite substantial dispersion among larger associations. Molecular characterization of small and large gametocyst pools recovered identical SSU rRNA sequences, indicating that the observed variability occurs within the same species. These results reveal substantial reproductive morphometric plasticity in *G. polymorpha* and highlight limitations of morphology alone for species delimitation in gregarines.

## 1 Introduction

Morphological stability has long been implicitly assumed in the taxonomy of many gregarines, despite increasing evidence that several developmental and reproductive traits may exhibit considerable plasticity. Gregarines are among the most diverse groups of apicomplexan parasites infecting invertebrates, yet their taxonomy remains notoriously difficult (Levine 1988; Clopton 2009). Species descriptions have traditionally relied on trophozoite morphology, syzygy organization, and gametocyst dimensions, frequently in the absence of molecular information (Levine 1988; Clopton 2009). Although these characters remain taxonomically informative, increasing evidence suggests that morphometric traits in gregarines may exhibit substantial phenotypic plasticity influenced by developmental stage, host physiology, nutritional status, and environmental conditions (Leander 2008; Simdyanov and Diakin 2013; Kolman et al. 2015).

Among reproductive stages, gametocyst morphology has often been considered comparatively stable and therefore useful for species characterization (Levine 1988; Clopton 2009). Nevertheless, marked variability in gametocyst size has occasionally been reported within single host populations or among closely related taxa (Watson 1916; Hoshide 1958; Clopton 2012), raising questions regarding the reliability of morphology alone for species delimitation. Likewise, the proportional organization of primite and satellite within syzygies has received relatively little quantitative attention despite its potential relevance for understanding reproductive biology and developmental organization (Simdyanov and Diakin 2013; Rueckert and Horák 2017).

Although morphological variability is frequently visible in published descriptions and micrographs of gregarines, most studies rely primarily on descriptive ranges and qualitative observations rather than formal analyses of morphometric distribution structure or proportional organization (Rueckert and Horák 2017). Consequently, the extent to which reproductive morphology reflects stable taxonomic characters, developmental plasticity, or intrinsic biological heterogeneity remains poorly understood. Quantitative approaches integrating morphometric distributions, proportional relationships, and molecular data may therefore provide a more informative framework for evaluating morphological variability in gregarines.

During the present study, we observed the coexistence of markedly different gametocyst sizes within a single host population of *Gregarina polymorpha* infecting larvae of *Tenebrio molitor* . Preliminary observations revealed both unusually small and exceptionally large gametocysts occurring under apparently homogeneous laboratory conditions. This raised several non‐exclusive hypotheses, including extreme intraspecific morphometric plasticity, developmental or physiological heterogeneity within a single species, or the coexistence of cryptic lineages indistinguishable through conventional morphology alone.

To investigate whether the marked variability observed in gametocyst morphology could be related to variability already present during syzygy formation, we conducted a quantitative morphometric analysis of 100 gametocysts and 100 independent syzygies. Because direct developmental tracking between individual syzygies and the gametocysts subsequently produced is not currently feasible in gregarines, both datasets were analyzed independently at the population level. In addition, preliminary molecular analyses were performed to evaluate whether morphometric extremes corresponded to distinct taxa or instead reflected variability within *G. polymorpha* .

## 2 Materials and Methods

### 2.1 Sampling

The study was conducted using naturally infected larvae of *Tenebrio molitor* maintained in our insectary at 25°C under a 12 h:12 h light–dark cycle. Wheat bran was provided as both substrate and food source, while humidity was maintained by adding carrot slices, which were replaced every two days. Because *T. molitor* larvae are frequently co‐infected by multiple gregarine species, only a colony infected exclusively with *G. polymorpha* was selected for the present study. Species identification was based on trophozoite morphology (Clopton et al. 1992) following the dissection and microscopic examination of 30 randomly selected larvae from the colony.

A total of 100 syzygies (paired gamonts) and 100 independent gametocysts were selected for morphometric analysis. Larvae dissections were performed by cutting off the posterior end of each larva and carefully extracting the intestinal tract, which was subsequently immersed in a drop of 0.9% NaCl solution on a microscope slide. Only the midgut region, extending from the stomach to the insertion site of the Malpighian tubules, where gregarines were most abundant, was retained for examination. The midgut was opened by making 12–15 transverse incisions with a scalpel in order to release the gregarines.

Gametocysts were analyzed independently from syzygies and were randomly collected from larval frass obtained by maintaining 50 infected larvae in a 14 cm diameter Petri dish for 24 h without substrate. To facilitate frass collection while providing food, only a few carrot slices were provided. After 24 h, 10 mL of physiological saline solution was added to the frass, and the Petri dish was maintained under gentle agitation for 1 h. Subsequently, 100 individual gametocysts were randomly selected for morphometric analysis. Each gametocyst was measured only once, and because it is currently not possible to determine which gametocyst originated from a particular syzygy, gametocysts and syzygies were treated as independent population‐level samples rather than paired developmental observations.

### 2.2 Morphometric Analysis

Morphometric analyses were performed using an Optika CP‐12 Pro digital camera (OPTIKA, Italy) coupled to either an Olympus CX41 light microscope or an Olympus SZ61 stereomicroscope (Olympus). For gametocysts, maximum length, width, and estimated area were recorded. For syzygies, measurements were independently obtained for primites and satellites, including length, width, estimated area, total syzygy area, and primite/satellite area ratio (P/S). Estimated areas were calculated assuming circular or elliptical geometry depending on specimen morphology. All morphometric measurements were performed using ProView digital image analysis software (OPTIKA, Italy).

### 2.3 PCR and Sequencing

To evaluate whether the marked morphometric differences observed between small and large gametocysts were associated with molecular differentiation, molecular characterization was performed using two independent gametocyst pools representing morphometric extremes. Pools were selected according to the distribution of gametocyst area measurements obtained from the analyzed population (Figure S1). A total of 20 small gametocysts and 20 large gametocysts representing the lower and upper extremes of the morphometric distribution were manually isolated for PCR and sequencing. Individual gametocysts selected for molecular analyses were isolated one by one under stereomicroscopic observation using a micropipette fitted with a 100 μL tip adjusted to a final volume of 20 μL, and subsequently transferred into sterile Eppendorf tubes. The gametocysts were then pelleted at 14,000 g for 30 s. DNA of each pool was extracted using the Speedtools tissue DNA extraction kit (Biotools B&M Labs, Spain). For PCR amplification, 5 μL of the final DNA elution volume (100 μL) was used as template. Amplification of the SSU rRNA gene was performed using the primer pair GRF (5′‐CGGTAATTCCAGCTCCAAT‐3′) and GRR (5′‐TGACTTGCGCTTACTAGGA‐3′). These primers were based on the universal apicomplexan primer pair designed by Nocciolini et al. (2018), targeting conserved regions located upstream of the V4 and downstream of the V8 hypervariable regions of the 18S rRNA gene. However, the reverse primer was slightly modified by replacing the first guanine at the 3′ end with adenine after BLASTn analyses revealed mismatches at this position in several gregarine sequences, potentially affecting primer annealing efficiency and specificity. The PCR was performed in 50 μL of reaction volume constituted of: 5 μL of DNA, 1 μL of both forward and reverse primers (10 mM), 1 μL of BIOTAQ DNA Polymerase (Bioline, United Kingdom), 3 μL of MgCl_2_ solution (50 mM), 0.5 μL of dNTP mix (100 mM), 5 μL of reaction buffer, and 33.5 μL of nuclease‐free water. The PCR was run in a Q‐Cycler 96 thermocycler (Hain Lifesciences) using the following conditions: an initial denaturation step at 94°C for 3 min, followed by 35 cycles of: a denaturation step at 94°C for 45 s, an annealing step at 55°C for 45 s, and an extension step at 70°C for 45 s. A final extension step at 72°C for 5 min was used. PCR products were visualized by electrophoresis in a 1% agarose gel to confirm expected size (~950 bp). Amplicons were sequenced in both directions in 10 μL reactions using Big Dye chemistries and an ABI 3730xl sequencer analyzer (Applied Biosystems, Foster City, CA, USA). Raw sequences were examined with ChromasPro software (Technelysium Pty Ltd., Australia) to generate consensus sequences. These sequences were compared with reference sequences deposited at the National Center for Biotechnology Information (NCBI) using the BLAST tool (Altschul et al. 1990).

### 2.4 Statistical Analyses

Statistical analyses were performed in R (R Core Team 2026). Descriptive statistics included mean, median, minimum and maximum values, coefficients of variation and distribution analyses. Normality was evaluated using the Shapiro–Wilk test. Morphometric heterogeneity of gametocysts was explored using histograms, density plots and Hartigan's dip test for unimodality. Relationships between primite and satellite areas were assessed using Pearson correlation, ordinary least squares regression and Standard Major Axis (SMA) regression.

## 3 Results

### 3.1 Morphometric Variability of Gametocysts

**FIGURE 1:**
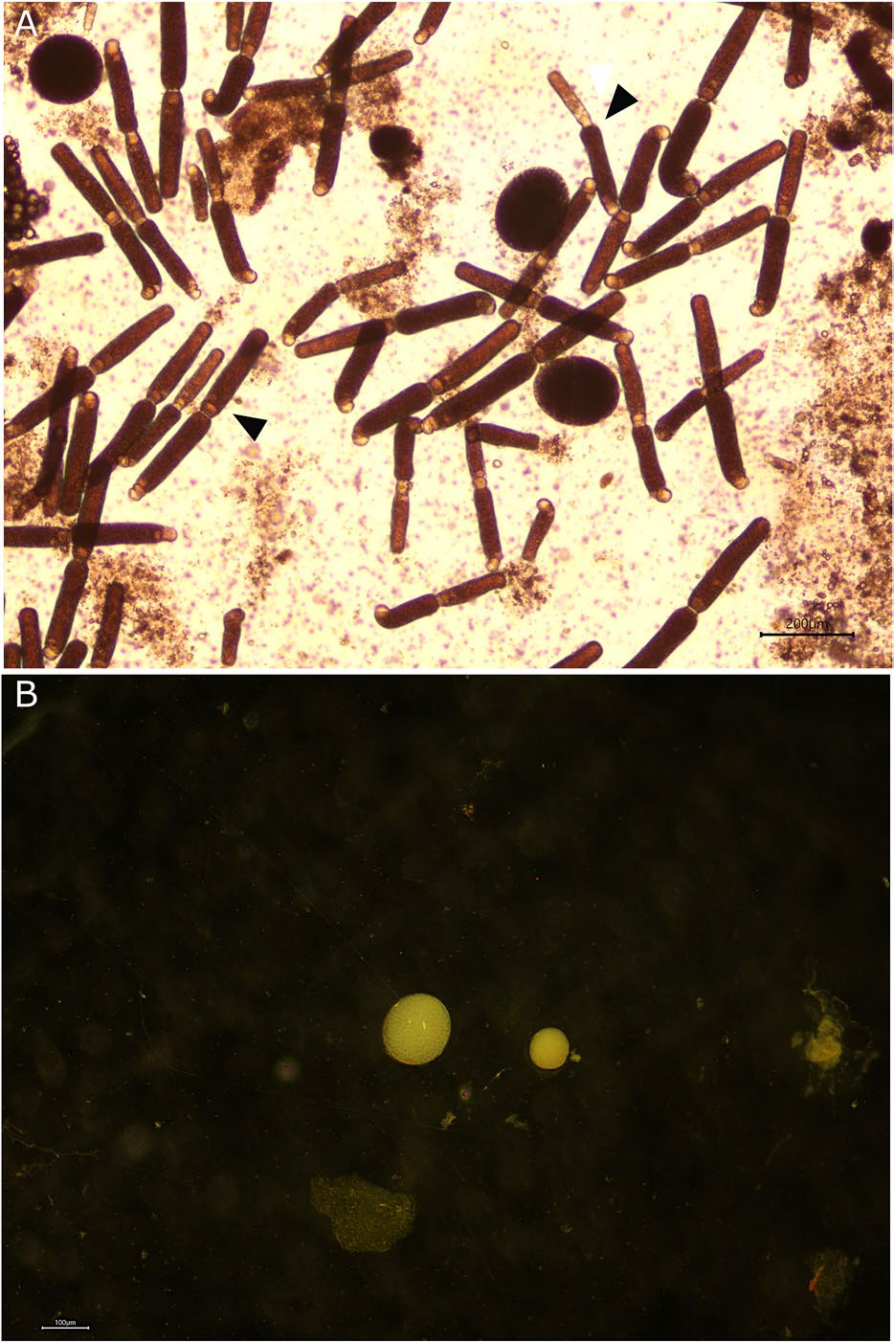
Representative micrographs illustrating the morphological variability observed in reproductive stages of Gregarina polymorpha. (A) Syzygies showing variation in overall size and proportional organization between primite and satellite gamonts. The upper arrowhead marks a representative asymmetric syzygy, whereas the lower arrowhead marks a representative symmetric syzygy. Micrograph obtained at 100× magnification. (B) Representative small and large gametocysts illustrating the marked size variability observed within the analyzed population. Micrograph obtained at 45× magnification using a stereomicroscope. Scale bars: 200 μm (A) and 100 μm (B).

Considerable variability was observed in both syzygy organization and gametocyst morphology within the analyzed population of *G. polymorpha* (Figure 1). Syzygies displayed marked differences in overall size and proportional organization between primite and satellite gamonts, including both symmetric and asymmetric associations. Likewise, gametocysts ranged from unusually small to exceptionally large forms.

The analyzed gametocyst population exhibited substantial morphometric variability (Table 1). Estimated gametocyst area ranged from 4850 to 30,844 μm^2^, representing more than a six‐fold difference within the sampled population. The coefficient of variation reached 31.3%, indicating pronounced heterogeneity in gametocyst size.

**TABLE 1:** Summary of morphometric parameters obtained from gametocysts and syzygies of *Gregarina polymorpha* infecting *Tenebrio molitor* larvae.

Structure	Parameter	Median	Mean	Min	Max	CV (%)	Distribution
Gametocysts	Area (μm^2^)	21,275	20,144	4850	30,844	31.3	Non‐normal
Syzygies	P/S ratio	1.004	1.143	0.558	3.553	—	Non‐normal

*Note:* Gametocyst measurements correspond to estimated area values, whereas syzygy measurements correspond to primite/satellite area ratios (P/S). Normality was assessed using the Shapiro–Wilk test.

**FIGURE 2:**
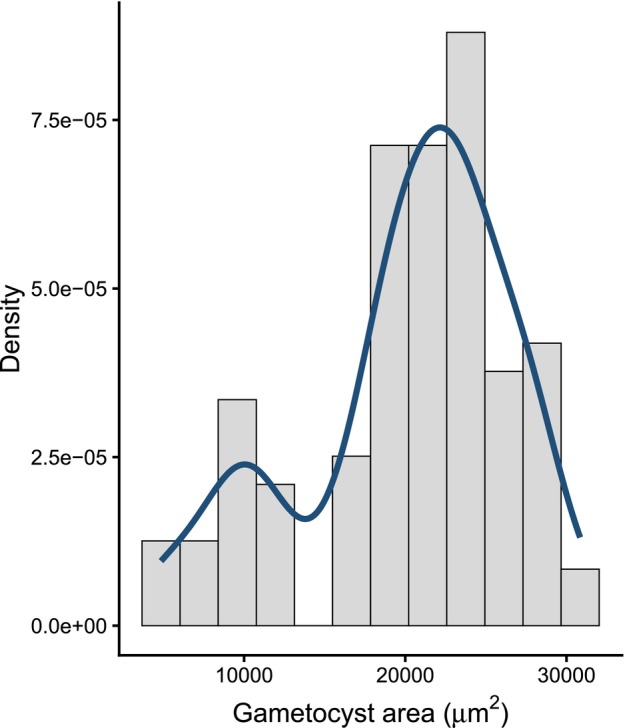
Histogram and kernel density distribution of gametocyst area in Gregarina polymorpha. The distribution is dominated by intermediate size classes but includes a subset of unusually small gametocysts contributing to the overall morphometric heterogeneity of the analyzed population.

The distribution of gametocyst areas significantly departed from normality (Shapiro–Wilk test, *p* < 0.001). Histogram and density analyses revealed a dominant size class centered approximately between 18,000 and 26,000 μm^2^, together with a smaller subset of unusually reduced gametocysts (Figure 2). Density plots revealed evident morphometric heterogeneity associated with the presence of these smaller morphotypes. However, Hartigan's dip test did not detect statistically significant deviation from unimodality (D = 0.035, *p* = 0.48).

### 3.2 Morphometric Relationships Within Syzygies

**FIGURE 3:**
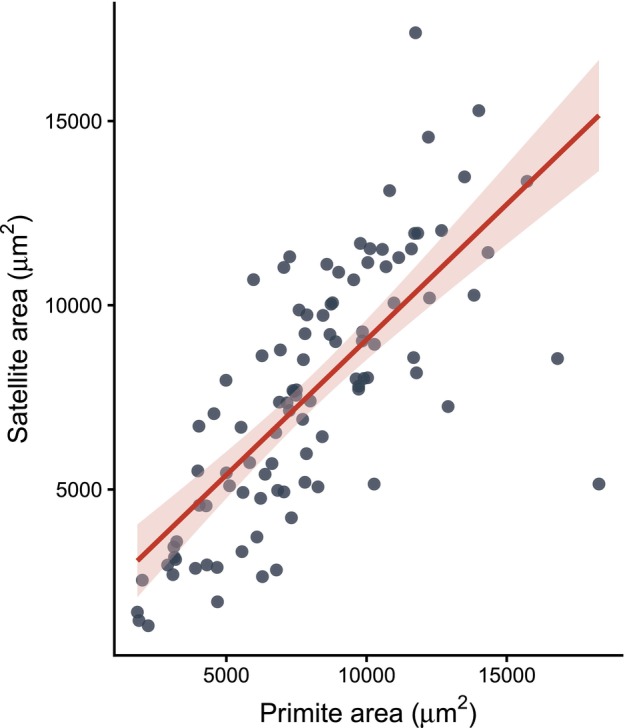
Relationship between primite and satellite areas in syzygies of Gregarina polymorpha. The fitted line corresponds to ordinary least squares linear regression. Increasing dispersion among larger syzygies indicates greater variability in proportional organization at larger sizes.

Morphometric parameters of syzygies are also summarized in Table 1. Primite and satellite areas showed a strong positive correlation (Pearson's *r* = 0.726, *p* < 0.001), indicating that larger primites are generally associated with larger satellites (Figure 3). Linear regression analyses revealed a significant positive relationship between primite and satellite area (*R* ^2^ = 0.53, *p* < 0.001). Scatterplot visualization demonstrated that dispersion increased progressively among larger syzygies, whereas smaller associations remained comparatively more proportional and compact. Several large syzygies deviated substantially from the central regression trend, indicating increased variability in proportional organization at larger sizes.

Standard major axis regression demonstrated scaling patterns consistent with isometry, with an estimated slope of 1.01 (95% CI = 0.88–1.16). Thus, despite the substantial variability observed among individual syzygies, primite and satellite growth remained globally proportional across the analyzed size range. The primite/satellite ratio (P/S) ranged from 0.558 to 3.553 (Table 1). The median ratio was close to unity (1.004), indicating that most syzygies consisted of relatively balanced associations. Nevertheless, the distribution was strongly right‐skewed and significantly departed from normality (Shapiro–Wilk test, *p* < 0.001).

**FIGURE 4:**
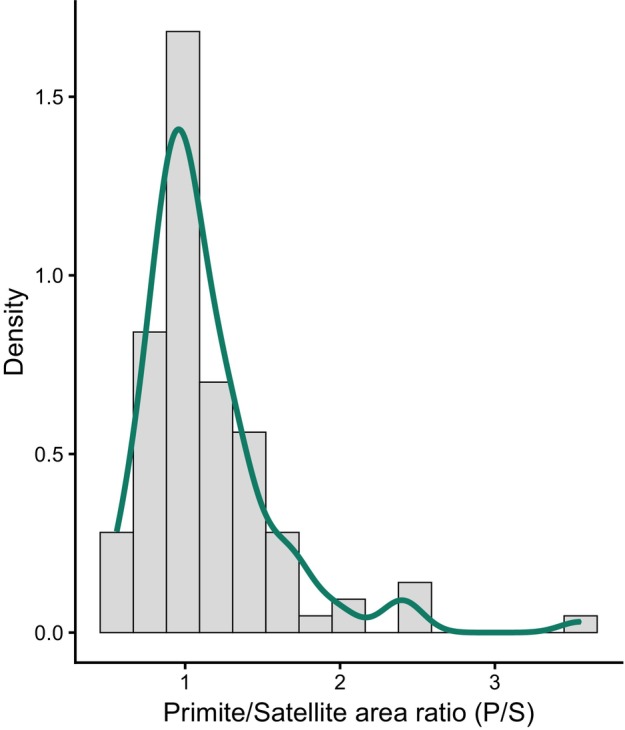
Histogram and kernel density distribution of the primite/satellite area ratio (P/S) in syzygies of Gregarina polymorpha. Most syzygies cluster around proportional associations (P/S≈1), whereas strongly asymmetric associations occur less frequently and generate a markedly right‐skewed distribution.

Histogram and density analyses of the P/S ratio revealed a dominant peak centered around proportional associations (P/S≈1), accompanied by a long right tail corresponding to increasingly asymmetric associations (Figure 4). Occasional highly asymmetric syzygies were therefore present despite the overall tendency toward proportional organization.

### 3.3 Molecular Characterization of Gametocyst Pools

SSU rRNA sequences obtained independently from the pools of small and large gametocysts were identical. BLASTn analyses identified both sequences as Gregarina polymorpha. Because no sequence variation was detected between pools, a representative sequence was deposited in GenBank under accession number PZ458080.

These results indicate that the marked morphometric differences observed between small and large gametocysts do not correspond to distinct molecular lineages and instead occur within the same species.

## 4 Discussion

The present study demonstrates substantial morphometric variability in both gametocysts and syzygies of Gregarina polymorpha. Gametocyst area varied more than six‐fold within a single host population, and the distribution departed significantly from normality because of the presence of a subset of unusually small individuals. However, Hartigan's dip test provided no statistical evidence for discrete morphotypes. In addition, SSU rRNA sequences obtained from pools representing the smallest and largest gametocysts were identical, indicating that the observed morphometric extremes belong to the same species rather than representing distinct molecular lineages.

These findings support the interpretation that considerable reproductive morphometric plasticity may occur within a single population of *G. polymorpha* . Several biological mechanisms could account for the observed variability. Differences in developmental stage, host physiological condition, parasite density, or local resource availability have all been proposed as potential sources of phenotypic variation in gregarines (Leander 2008; Simdyanov and Diakin 2013). Because all larvae examined in the present study were maintained under homogeneous laboratory conditions, environmental variation is unlikely to be the sole explanation. Instead, intrinsic developmental or physiological differences among parasites may contribute substantially to the observed morphometric heterogeneity. Although cryptic diversity cannot be completely excluded on the basis of a single molecular marker, the identical SSU rRNA sequences obtained from both morphometric extremes strongly support the conclusion that the observed variation represents intraspecific variability rather than distinct species.

Although not directly evaluated in the present study, the observed variation in gametocyst size may also have functional consequences. Larger gametocysts have been shown to produce higher numbers of oocysts in other gregarine species (Borengasser and Clopton 2019), suggesting that differences in gametocyst size could influence reproductive output and transmission potential. Whether the unusually small gametocysts observed here represent physiologically constrained individuals, less productive reproductive stages, or alternative developmental trajectories remains unknown and deserves further investigation.

The morphometric analysis of syzygies provides additional insight into reproductive organization in *G. polymorpha* . Primite and satellite areas were strongly positively correlated, and standard major axis regression indicated scaling patterns consistent with approximate isometry across the analyzed size range. Nevertheless, larger syzygies exhibited greater dispersion in primite–satellite proportions than smaller associations, indicating that proportional organization becomes increasingly variable as size increases. Although the mechanisms responsible for this pattern were not investigated, it could reflect greater developmental flexibility or physiological differences between associated gamonts during growth.

Taken together, these results highlight the limitations of relying exclusively on morphology for species delimitation in gregarines. Despite the remarkable variation observed in reproductive morphology, molecular characterization indicated that the analyzed morphotypes belong to a single species. This finding reinforces previous recommendations that quantitative morphometric analyses should be interpreted together with molecular data when assessing species boundaries in gregarines (Rueckert and Horák 2017).

Several limitations should nevertheless be acknowledged. Molecular characterization was restricted to pooled gametocysts representing the extremes of the morphometric distribution and relied on a single genetic marker (SSU rRNA), which may not detect more subtle population‐level genetic differentiation. Furthermore, all specimens originated from a single laboratory‐maintained host colony and may therefore not encompass the full range of variation present in natural populations. Future studies combining additional molecular markers, broader geographic sampling, and experimental analyses of parasite development will be valuable for identifying the mechanisms responsible for the remarkable reproductive morphometric plasticity observed in *G. polymorpha* .

## Supporting information


**Figure S1:** Distribution of gametocyst areas measured in the analyzed population of *G. polymorpha* infecting *T. molitor* larvae. Gametocysts are ordered according to increasing area values. Red and blue points indicate the specimens selected for molecular characterization of the small and large morphometric pools, respectively. Both pools yielded identical SSU rRNA sequences despite the marked morphometric differences observed between them.

## Data Availability

The data that support the findings of this study are available from the corresponding author upon reasonable request.
