# Supplementary figures and images for: Reproductive Morphometric Plasticity in Gregarina polymorpha Infecting Tenebrio molitor Larvae
Source: J Eukaryot Microbiol. 2026 Jul 31;73(5):e70110. doi: 10.1111/jeu.70110 (PMC13425064; doi:10.1111/jeu.70110)

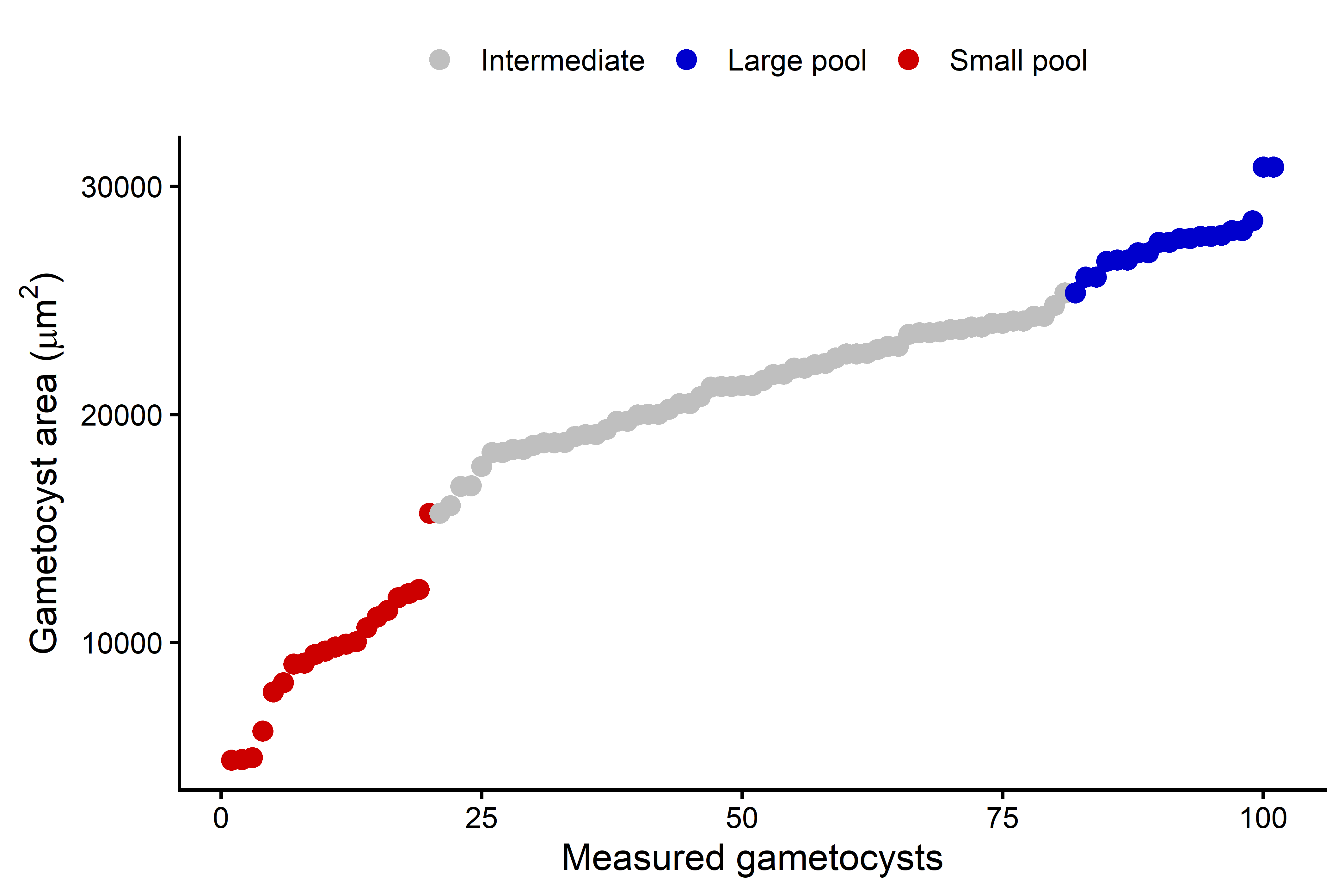

Supplement: Supplementary file 1 — Figure S1: Distribution of gametocyst areas measured in the analyzed population of G. polymorpha infecting T. molitor larvae. Gametocysts are ordered according to increasing area values. Red and blue points indicate the specimens selected for molecular characterization of the small and large morphometric pools, respectively. Both pools yielded identical SSU rRNA sequences despite the marked morphometric differences observed between them. [file JEU-73-e70110-s001.tiff]
